# On-surface synthesis of non-benzenoid conjugated polymers by selective atomic rearrangement of ethynylarenes†

**DOI:** 10.1039/d2sc04722e

**Published:** 2022-12-20

**Authors:** Alejandro Jiménez-Martín, Federico Villalobos, Benjamin Mallada, Shayan Edalatmanesh, Adam Matěj, Juan M. Cuerva, Pavel Jelínek, Araceli G. Campaña, Bruno de la Torre

**Affiliations:** a Regional Centre of Advanced Technologies and Materials, Czech Advanced Technology and Research Institute (CATRIN), Palacký University Olomouc Olomouc 783 71 Czech Republic bruno.de@upol.cz; b Faculty of Nuclear Sciences and Physical Engineering, Czech Technical University in Prague Brehova 7 Prague 1 115 19 Czech Republic; c Departamento de Química Orgánica, Universidad de Granada (UGR), Unidad de Excelencia de Química UEQ, C. U. Fuentenueva Granada 18071 Spain aracelig@ugr.es; d J. Department of Physical Chemistry, Faculty of Science, Palacký University Olomouc 78371 Czech Republic; e Institute of Physics, Czech Academy of Sciences Prague 162 00 Czech Republic

## Abstract

Here, we report a new on-surface synthetic strategy to precisely introduce five-membered units into conjugated polymers from specifically designed precursor molecules that give rise to low-bandgap fulvalene-bridged bisanthene polymers. The selective formation of non-benzenoid units is finely controlled by the annealing parameters, which govern the initiation of atomic rearrangements that efficiently transform previously formed diethynyl bridges into fulvalene moieties. The atomically precise structures and electronic properties have been unmistakably characterized by STM, nc-AFM, and STS and the results are supported by DFT theoretical calculations. Interestingly, the fulvalene-bridged bisanthene polymers exhibit experimental narrow frontier electronic gaps of 1.2 eV on Au(111) with fully conjugated units. This on-surface synthetic strategy can potentially be extended to other conjugated polymers to tune their optoelectronic properties by integrating five-membered rings at precise sites.

## Abstract 2

Here, we report a new on-surface synthetic strategy to precisely introduce five-membered units into conjugated polymers from specifically designed precursor molecules that give rise to low-bandgap fulvalene-bridged bisanthene polymers.

## Graphical abstract

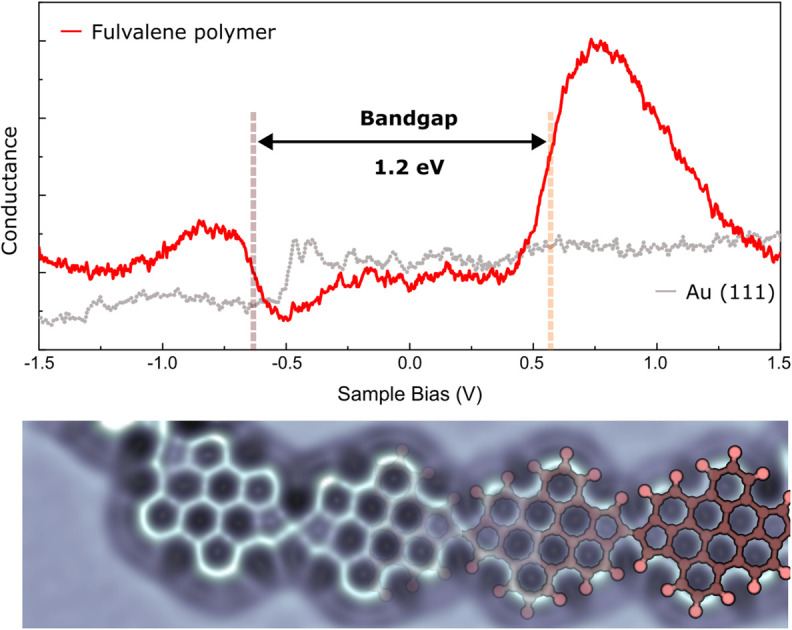

## Introduction

The ability to control their characteristics with rational chemical synthesis has made conjugated polymers excellent candidates for technological applications such as light emitting devices, solar cells, organic field-effect transistors, photocatalysts and biosensors.^1,2^ These semiconductors and synthetic metals have been generated from a wide range of organic precursors comprising heterocyclic compounds as well as non-benzenoid polycyclic hydrocarbons.^3^ The successful synthesis of these non-benzenoid compounds has recently provided profound insights into the electronic properties of antiaromatic and/or open-shell systems.^4–9^ For example, these antiaromatic compounds have demonstrated higher charge carrier mobility than their aromatic counterparts,^10^ although the interpretation of these properties is still unclear.^11,12^ Unfortunately, conventional wet synthesis of conjugated polymers containing non-benzenoid compounds is difficult in the case of unstable final compounds due to their inherent low solubility, high intrinsic reactivity and the occurrence of undesirable structural defects^13^ that prevent complete control of the molecular structure.

On-surface synthesis^14,15^ under ultra-high vacuum (UHV) conditions is a promising strategy for synthetizing non-benzenoid compounds. This approach has proven to be ideal for fabricating (macro)molecular architectures with atomic precision and tailored electronic properties.^16^ The rational design of precursor molecules and the stabilization offered by single-crystal substrates allow the engineering of specific products, enabling the fine-tuning of their structural and electronic properties,^15^ the fabrication of intrinsically reactive molecular structures^7–9,17^ and the investigation of rearrangement reactions^18–23^ of particular interest for non-benzenoid π-extended nanostructures.^24^ It therefore provides unique opportunities to address the scientific challenge of fabricating well-defined conjugated polymers incorporating non-benzenoid components, with the goal of designing chemically robust, low bandgap polymers. Nevertheless, there are only a few reports discussing the on-surface formation of non-benzenoid moieties,^18,25–29^ sometimes in conjugated polymers.^8,21,30–35^ Most of them have utilized strategies based on oxidative ring closure,^8,36^ bond rotation^37^ or the use of molecular precursors with embedded 5-membered rings.^38,39^ Therefore, it is highly desirable to provide new strategies that allow the controlled formation and detailed analysis of such non-benzenoid molecules.

**Fig. 1:**
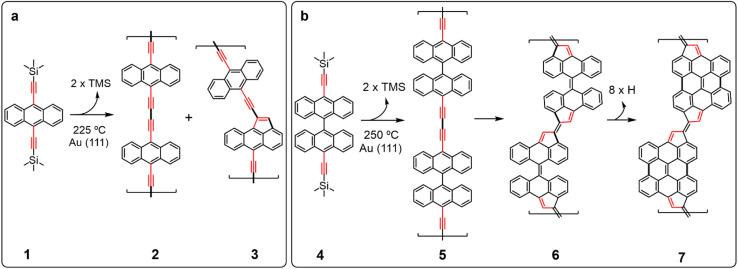
Scheme of the reaction sequence of (a) anthracene and (b) bianthracene based precursors after being deposited on Au(111) and annealed up to 225 °C and 250 °C, respectively, to produce conjugated polymers incorporating non-benzenoid units.

Here, we exploited the atomic rearrangement of ethynylarene to cyclopenta-fused polycyclic aromatic hydrocarbons, to give rise to low bandgap fulvalene-bridged bisanthene polymers on an atomically flat Au(111) surface. To this end, we synthetized 9,10-bis(trimethylsilyl)ethynylanthracene and 10,10′-bis((trimethylsilyl)ethynyl)-9,9′-bianthracene as precursors (1 and 4 in Fig. 1, respectively). Each of them was intentionally designed with a dual purpose: (i) to form one-dimensional diethynyl-bridged polymers after homo-coupling of terminal alkynes (Glaser-like coupling) on the surface;^40^ and (ii) to favor the selective atomic rearrangement of the linker giving rise to non-benzenoid units.^41^ Furthermore, endowing precursors 1 and 4 with trimethylsilyl (TMS) groups allows chemical protection of the compounds and the *in situ* deprotection of such terminal alkynes on-surface under UHV conditions. This strategy offers an alternative to the use of unstable or highly-reactive monomers.^42–44^ Using scanning tunneling microscopy and spectroscopy (STM and STS), in combination with non-contact atomic force microscopy (nc-AFM) and density functional theory (DFT) calculations, we analyzed the chemical structure and electronic properties of the reaction products. Importantly, overall, we reveal the successful formation of fulvalene-bridged bisanthene conjugated polymers that exhibit a low bandgap of a measurable value of 1.2 eV.

## Results and discussion

Fig. 1 shows the chemical structure of the precursor molecules 1 and 4, which we have used in this study. 1 and 4 were synthesized in solution from commercially available 9,10-dibromoanthracene and 10,10′-dibromo-9,9′-bianthracene, respectively, *via* double Sonogashira coupling with trimethylsilylacetylene (see ESI† for details of the synthesis). The corresponding unprotected terminal alkynes 9,10-diethynylanthracene and 10,10′-diethynyl-9,9′-bianthracene were also synthesized in solution by treatment of 1 and 4 with tetrabutylammonium fluoride. However, we observed that these compounds tend to react or decompose under ambient conditions. Besides, thermo gravimetric analysis (TGA) shows a gradual decomposition of protected 4 at temperatures above the evaporation threshold (Fig. S1†). For this reason, we decided to keep the trimethylsilyl protecting groups and perform *in situ* deprotection prior to the on-surface polymerization.

**Fig. 2:**
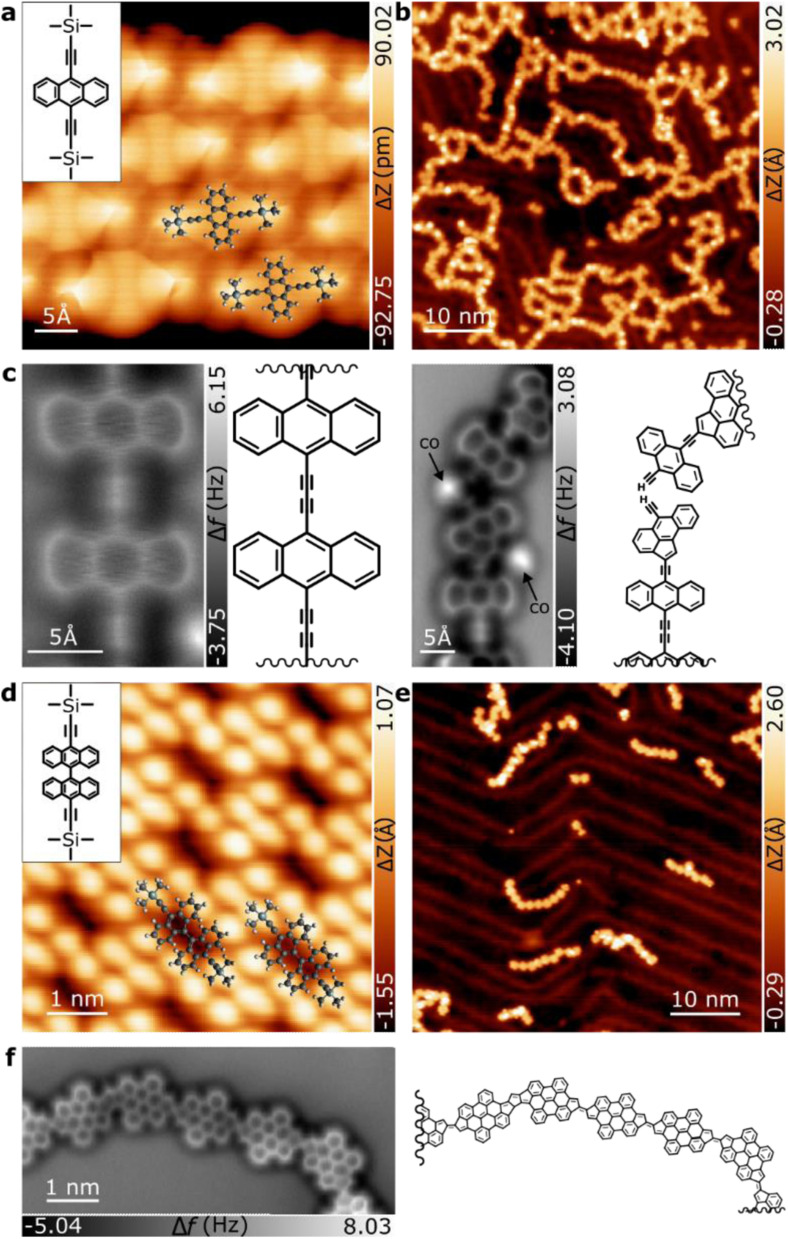
On-surface synthesis of fulvalene-bonded bisanthene polymers. (a) STM topographic overview with a superimposed model of precursor (1) (top left inset) upon deposition at RT on Au(111) (0.05 V, 0.01 nA). (b) STM topographic overview after thermal annealing at 225 °C (0.1 V, 0.01 nA). (c) nc-AFM detailed images of the one-dimensional molecular structures of (b) including their respective model. The images show the presence of diethynyl bonds and ethynyl-pentagon joints. (d) STM topographic overview with a model of precursor (4) (top left inset) after deposition at RT on Au(111) (−2 V, 0.05 nA). (e) STM topographic overview of the sample after annealing at 250 °C (0.1 mV, 0.02 nA). (f) High-resolution nc-AFM image of a fulvalene-bridged bisanthene polymer and the corresponding model of the polymer.

Sublimation of 1 under UHV conditions on clean Au(111) maintained at room temperature gives rise to self-assembled molecular islands of anthracene units with their remaining TMS groups, as depicted by the STM and nc-AFM images (Fig. 2a, inset) and in perfect agreement with previous reports on Ag(111).^45^ The STM images after sample annealing at 225 °C (*cf.*Fig. 2b) reveal that polymerization of 1 has been triggered, leading to the formation of one-dimensional molecular structures on the surface. In the close-up nc-AFM image recorded with a functionalized CO-tip (Fig. 2c), polymer units were resolved. These consist of anthracene units coupled by diethynyl motifs, confirming the formation of 2. The two triple bonds are unambiguously distinguishable as bright dots, which is consistent with recent observations on synthetized ethynylene-bridged anthracene polymers^46^ and poly(*p*-phenylene ethynylene) molecular wires on Au(111).^47^ Interestingly, compared to the reported ethynylene-bridges, diethynyl units increase the electronic bandgap from 1.5 eV^46^ to approximately 1.9 eV (see Fig. S2†). In addition, intramolecular rearrangement of 2 is frequently observed, and a significant number of five-membered rings are found in the linkers, giving rise to structure 3, as shown in Fig. 2c, right. From the STM/AFM images, we inferred that residual hydrogen from the environment chemically passivates the alkynes at the termini of the polymers. The fact that these non-benzenoid units are consistently found in the linkers indicates that they all originated by arrangement of the ethynylene moiety. Indeed, it is clear that along the bridged unit, the 5-membered ring can be found to be coupled to an ethynylene segment, as can be inferred by the presence of the triple bond in the nc-AFM images (*cf.*Fig. 2c, right). Thus, the linear anthracene-diethynyl-anthracene backbone reacts to form five-membered rings by thermally induced ethynylarene rearrangement to cyclopenta fused anthracene, as previously described.^48^ This finding suggests not only the possibility of tuning the polymer bandgap by introducing the diethylene linker, but a mechanism for inducing the formation of five-membered rings by selective atomic rearrangement.

To strengthen our hypothesis and investigate the generality of the ethynylarene rearrangement to cyclopenta fused polycyclic aromatic hydrocarbons, we synthesized compound 4 (Fig. 1) and studied its polymerization reaction on the Au(111) surface with the aim of obtaining low bandgap conjugated polymers. Pleasingly, *in situ* alkyne deprotection and Glaser-like coupling on the Au(111) surface leading to bisanthene-based conjugated polymers also worked with 4, albeit at higher temperatures (see Fig. S3†). Similarly, STM images of islands formed by bianthracene 4 display bright rounded features due to the bulky TMS protecting groups (Fig. 2d). A first step of annealing 4 on Au(111) to *T* = 250 °C for 30 minutes triggers the polymerization of 4 leading to the formation of one-dimensional molecular structures on the surface (*cf.*Fig. 2e). The chains have an average length of 9.6 nm (see Fig. S3†) and are composed of straight and zig-zag segments, which can be found in *cis*- or *trans*-configurations depending on the bonding between monomers (see Fig. S4† for more details). They do not exhibit protrusions, indicating successful planarization of the units. Nc-AFM imaging with a functionalized CO-tip^49^ was used to reveal the chemical structure of the polymer. Its skeleton was unambiguously resolved (Fig. 2f), confirming that it is composed of a sequence of bisanthene monomers with fused fulvalene bridges (7). Notably, the reaction is highly selective and only minority concomitant defects are detected for submonolayer coverage. The calculated bond dissociation energy (BDE) of the reaction-participant radical positions for anthracene (2), bianthracene (5), and bisanthene polymers in the gas phase (Fig. S5†) shows that the dissociation required to initiate the cyclo-pentafused rearrangement yields significantly different values for anthracene and bianthracene polymers. The formation of pentagon moieties is less energetically favorable for anthracene polymers in the gas phase by 5.2 kcal mol^−1^, indicating selective initiation of the atomic rearrangement.

**Fig. 3:**
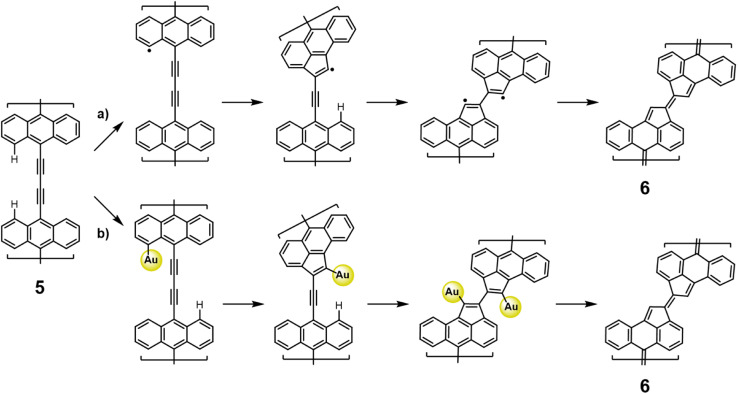
Proposed reaction mechanism giving rise to fulvalene units. (a) Traditional, and (b) on-surface proposed ethynylarene rearrangement to cyclopenta fused polycyclic aromatic hydrocarbons. An initial hydrogen abstraction is followed by oxidative ring closure and π-electron reconfiguration, while individual gold atoms may stabilize transient radicals.

The absence of 1,3-butadiyne traces suggests that they all converted to fulvalene bridges. In principle, the rearrangement of ethynylarene to cyclopenta fused polycyclic aromatic hydrocarbons may proceed through any of the five distinct sequences of elementary reactions that differ in the temporal order of ring closure and hydrogen migration/transfer. Our findings provide no direct evidence of a preference for any temporal order but taking into account the capability of gold surfaces to activate aromatic CH bonds,^50^ the generation of an aryl radical followed by 5-*endo-dig* radical cyclization^51^ and subsequent passivation of the generated radical seems to be the most simple and plausible mechanism considering the reported thermal rearrangement of ethynylarenes in solution (*cf.*Fig. 3a).

It is notable that the proposed mechanism at the surface may involve the activity of individual gold adatoms^52,53^ to passivate the emerging radicals, which may ultimately stabilize with residual hydrogen, highlighting the catalytic role of single atoms at the surface (see Fig. 3b). Unfortunately, no intermediate structures (5 and 6 in Fig. 1) could be identified to support a rearrangement mechanism. Importantly, all of our efforts to generate diethynylene-bridged bisanthene polymers by lowering the reaction temperature on the Au(111) substrates failed, clearly confirming that the formation of the five-membered rings is highly efficient (see Fig. S3†). We also tested the possibility of growing Glaser polymers (1 and 4) on Ag(111). However, the surface showed low reactivity for such coupling. This is probably due to the strong molecule–substrate interaction, leading to low molecular diffusion, which is a prerequisite for efficient on-surface chemistry.

**Fig. 4:**
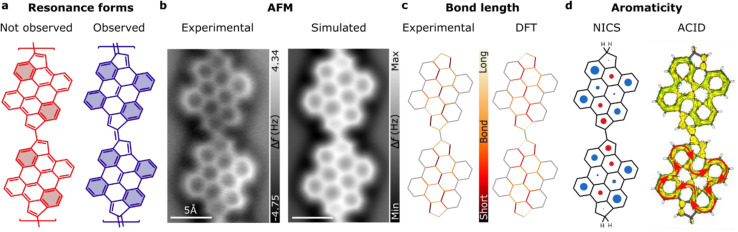
Resonance and bond analysis of fulvalene-linked polymers. (a) Proposed resonance structures of bisanthene-fulvalene polymers. In blue (red) are the observed (not-observed) resonance forms of the fulvalene-based polymers on the surface (Clar's sextets are highlighted). (b) Experimental (left) and simulated (right) nc-AFM images of fulvalene-bridged one-dimensional structures. (c) Experimental (left) and DFT (right) bond length analysis for a bisanthene-fulvalene dimer. (d) Calculated nucleus-independent chemical shift (NICS) of a fulvalene dimer (left) and calculation of the induced current density (ACID) revealing the π-conjugation of the system (right). The diameter of the circle features corresponds to the qualitative aromatic/antiaromatic (blue/red) character of the ring (see Table in Fig. S6†).

Next, we examined the structural and electronic properties of the fulvalene-bridged bisanthene polymer 7, including the dominant resonance shape. In this regard, the rule of Glidewell and Lloyd^54^ provides a conceptual picture for predicting the resonance form in polycyclic aromatic hydrocarbons incorporating non-benzenoid units. According to this rule, a resonance structure with the smallest 4*n* + 2 groups, avoiding the formation of the smallest 4*n* groups, represents the most stable form or the one that contributes most to the resonance. Locally, the fulvalene bridge can exhibit two types of resonance forms, *i.e.*, the C–C bond connecting two pentagons can be single or double. From this hypothesis, at least two distinct resonance forms can be conceived, as illustrated in Fig. 4a. Application of Glidewell and Lloyd's rule shows that the structure associated with a double bond linker (blue color in Fig. 4a) should be the most stable, since the other option (red color in Fig. 4a) would imply the formation of four groups with 4π-electrons in the bisanthene moiety, which should be avoided according to the rule. Interestingly, the system stabilizes four Clar's sextets on the bisanthene unit (depicted in blue in Fig. 4a), the maximum number. To investigate this hypothesis, we performed bond order discrimination using nc-AFM with a CO-tip.^55^ High-resolution nc-AFM images of the fulvalene-bridged polymers (see Fig. 4b) show different bond lengths within the bisanthene unit, whose statistically average value is shown in the left panel of Fig. 4c (see Fig. S6† for details). The bond length analysis was qualitatively confirmed by DFT calculations (right panel of Fig. 4c). The analysis revealed that the variation of the bond distance in the polymer matches the π-resonance predicted by Glidewell and Lloyd's rule, further corroborating its validity on surfaces.

Although aromaticity is well understood for benzenoid compounds, the application of these concepts to non-benzenoid systems is not trivial. A better understanding of their “aromatic character” may lead to fruitful theoretical proposals and to the synthesis of novel non-benzenoid conjugated compounds on surfaces. In addition, the aromatic character of a given compound determines the relationship between its constituents and its chemical reactivity or electron delocalization energy.^56^ Therefore, it is interesting to analyze the degree of aromaticity and antiaromaticity of the bisanthene-fulvalene polymer. The left panel of Fig. 4d shows the calculated nucleus-independent chemical shift (NICS)^57^ of a bisanthene-fulvene dimer passivated by H_2_ at the edges to induce the double bond character of the C–C bond connecting two pentagons. Thus, the structure is closed shell since the potential radicals are quenched, at least for the dimer. NICS analysis revealed that the four benzenoid rings at the bisanthene kinks are clearly aromatic, while the central six-membered ring possesses values close to zero, typical of non-aromaticity, thus reproducing well the bond length analysis and corroborating the resonance form discussed above. On the other hand, in the pentagons of the fulvalene segment, we find positive values for the shielding tensor component (−σzz), which may indicate antiaromaticity. Qualitatively, however, those values are close to zero, which may indicate that the pentagons do not participate in the π-conjugation of the system. The deviation of conjugation pathway from zig-zag edge near the fulvalene rings effectively prolongs the conjugation path from 22 to 26 electrons, which explains the positive shielding on the benzene rings. The intensity of positive shielding above fulvene and the central benzene rings is much lower than that of proto-typical antiaromatic molecules, such as cyclobutadiene and pentalene, so they are considered non-aromatic (see Fig. S7† for the quantitative analysis of NICS calculation for the H and H_2_ terminated dimer and tetramer).

To gain further insight into the aromatic character of the polymer, we performed calculations of the anisotropy of the induced current density (ACID)^58^ (Fig. 4d right panel). The π-ACID (including only pz orbitals) shows a clear conjugation within each bisanthene unit including 26 π-electrons in total. The map reveals the interactions of ring currents of each individual ring, enhancing or suppressing the boundary with the neighboring ring, depending on their mutual orientations. Indeed, it reveals that there is aromatic π-conjugation within bisanthene units with the fulvalene units excluded. The ACID map shows the clockwise direction of the main current ring on the bisanthene periphery, revealing its aromatic character with all π-electrons involved in this current, in agreement with the 4*n* + 2 rule of aromaticity. We found that it is possible to include the fulvene moieties in the global ring current by passivating the dimer edges with H instead of H_2_ (see Fig. S8† for the quantitative analysis of ACID calculation for the H and H_2_ terminated dimer and tetramer). However, this leads to the single bond character of the C–C bond connecting the pentagons and an antiaromatic number of π-electrons, which goes against Glidewell and Lloyd's rule and the experimental observations. Conjugation through the linker shows a higher critical isosurface value for the double-bond linker (CIV 0.0395) than for the single-bond linker (CIV 0.0311, not shown), showing higher electron delocalization between the units. To conclude, the fulvalene polymer is aromatic with non-aromatic linkers, resulting in weak conjugation between units.

**Fig. 5:**
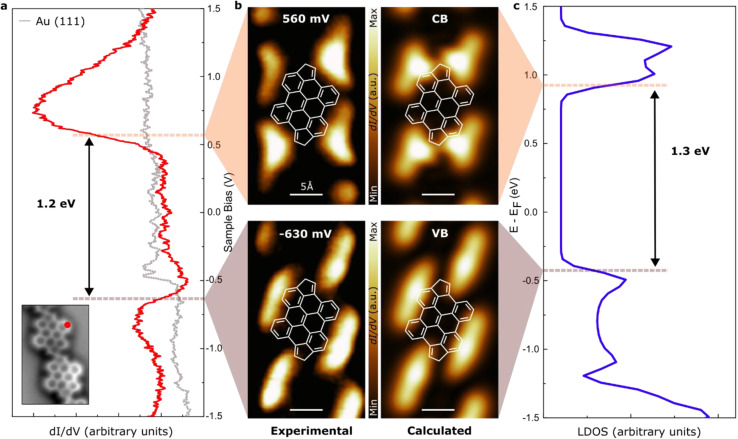
Electronic structure of fulvalene polymers. (a) d*I*/d*V* conductance spectra of the polymer acquired at the position marked with red in the inset image. The red profile features a gap corresponding to 1.2 eV. (b) Experimental constant current d*I*/d*V* maps (left) and calculated PDOS (right) at the conductance band (top) and valence band (down) onsets. (c) DFT calculated LDOS for fulvalene polymers in the gas phase featuring a bandgap of 1.3 eV.

Finally, to access the intrinsic electronic characteristics of the bisanthene-fulvalene polymers, we performed a set of scanning tunneling microscopy and spectroscopy experiments. A voltage-dependent differential conductance spectrum (d*I*/d*V vs. V*) acquired on 7 revealed peaks in the density of states (DOS) at −630 mV and 560 mV (Fig. 5a). Those peaks arise respectively from hybridization of the polymer valence and conduction bands (VB and CB) with the substrate. Spatial mapping of the d*I*/d*V* signal (d*I*/d*V* maps) at peak positions revealed characteristic features (Fig. 5b) that are well reproduced by the corresponding B3LYP-DFT-calculated d*I*/d*V* maps for an oligomer formed by 7 (Fig. 5c). The d*I*/d*V* map of the VB shows maxima at the lateral edge of the bisanthene moiety, with negligible charge density over the bridge (see Fig. 5b). The d*I*/d*V* map of the CB exhibits states on the empty spaces adjacent to the fulvene bridges (see Fig. 5b). Although DFT calculations could not qualitatively predict the magnitude of the intrinsic bandgap of the polymer^59^ (Fig. 5c), they described very well the character of the frontier orbitals of the VB and CB edges of the polymer (*cf.* Fig. S9†). In fact, the excellent agreement between experimental and simulated d*I*/d*V* maps validates the character of the frontier orbitals predicted by DFT. Thus, this results in a low bandgap of ∼1.2 eV on Au(111). It should be noted that the bandgap value obtained from STS measurements is typically reduced by an additional electron screening imposed by the proximity of a metallic surface with respect to the intrinsic bandgap of the gas-phase polymer.^60^ However, in this case, the perfect agreement between experimental and DFT values of the electronic bandgap indicates a low degree of hybridization of the fulvalene-polymer with the metal support.

## Conclusion

In conclusion, by studying the rearrangement of ethynylarene to cyclopenta fused polycyclic aromatic hydrocarbons on surfaces, we have introduced a novel synthetic strategy for the selective incorporation of five-membered rings into conjugated polymers. In particular, trimethylsilylacetylene protection/deprotection is a well-established strategy in solution-phase chemistry, which we believe also has great potential in the area of on-surface chemistry. The successful realization of bisanthene-fulvalene polymers is confirmed by STM and high-resolution nc-AFM analyses. We found that such a polymer exhibits a measurable low band gap of 1.2 eV and a closed-shell electron configuration on Au(111), as demonstrated by STS supported by DFT calculations. We anticipate that our new synthetic strategy will open avenues to fabricating highly demanded covalent polymers on surfaces incorporating non-benzenoid moieties, which are particularly attractive for organic photovoltaics, photodetectors, and ambipolar field-effect transistors.^61–63^

## Data availability

Data will be available on request. CCDC-2193258 contains the supplementary crystallographic data for this paper. More experimental details can be found in the ESI.†

## Author contributions

A. G. C. and B. T. conceived and designed the experiments. A. G. C and B. T. supervised the project and led the collaboration efforts. A. J.-M., B. M. and B. T. carried out the SPM experiments, obtained the data and performed on-surface reactions. F. V, J. M. C. and A. G. C. synthesized the precursors. The experimental data were analysed by A. J.-M. and B. T., and discussed by all the authors. S. E., A. M. and P. J. performed the theoretical calculations. The manuscript was written by A. J.-M., A. G. C and B. T. with contributions from all the authors.

## Conflicts of interest

There are no conflicts to declare.

## Supplementary Material

SC-014-D2SC04722E-s001
