# Supplementary material for: On-surface synthesis of non-benzenoid conjugated polymers by selective atomic rearrangement of ethynylarenes
Source: Chem Sci. 2022 Dec 20;14(6):1403–12. doi: 10.1039/d2sc04722e (PMC9906656; doi:10.1039/d2sc04722e)
Supplement: SC-014-D2SC04722E-s001 [file SC-014-D2SC04722E-s001.pdf]

Electronic Supplementary Material (ESI) for Chemical Science.
This journal is © The Royal Society of Chemistry 2022

Supplementary Information:

On-surface synthesis of non-benzenoid conjugated polymers by selective atomic rearrangement of ethynylarenes.

Alejandro Jiménez-Martín,[1,2,5†] Federico Villalobos,[3†] Benjamín Mallada,[1,4,5] Shayan Edalatmanesh,[1,4,5] Adam Matěj,[1,4,5] Juan M. Cuerva,[3] Pavel Jelínek,[1,5] Araceli G. Campaña,[3*] Bruno de la Torre,[1,5*]

1 Regional Centre of Advanced Technologies and Materials, Czech Advanced Technology and Research Institute (CATRIN), Palacký University Olomouc, 78371 Olomouc, Czech Republic.
2 Faculty of Nuclear Sciences and Physical Engineering, Czech Technical University in Prague, Brehova 7, 115 19 Prague 1, Czech Republic
3 Departamento de Química Orgánica, Universidad de Granada (UGR), Unidad de Excelencia de Química UEQ, C. U. Fuentenueva, 18071 Granada, Spain
4 Department of Physical Chemistry, Faculty of Science, Palacký University, 78371 Olomouc, Czech Republic.
5 Institute of Physics, Academy of Sciences of the Czech Republic, Prague, Czech Republic

*Correspondence to: aracelig@ugr.es (A.G.C), bruno.de@upol.cz (B.T.)

[†] These authors contributed equally to this work.

Contents

Methods
Figures S1 to S8
Synthesis of the materials
NMR spectra
MS spectra
Single crystal X-Ray crystallography
References

Methods

SPM experiments

The experiments were carried out in an ultra-high vacuum (UHV) system, with a base pressure below 5 x 10^{-10} mbar, hosting a low temperature (4.2 K) scanning tunneling microscope/non-contact atomic force microscope (Createc). Images were captured with a Pt/Ir tip attached to a qPlus sensor (resonant frequency ≈ 30 kHz; stiffness ≈ 1800 N m^{-1}). A bias voltage was applied to the sample.

Sharp metallic tips were obtained by gentle indentations on the bare surface. For AFM imaging, a single CO molecule is picked up by the tip-apex previously dosed on the cold sample (T 10 K).

The Au(111) substrate was prepared by standard cycles of Ar^+ sputtering and annealing. Molecular precursors **1** and **4** were thermally sublimated in UHV onto the clean Au(111) substrate (kept at room temperature) from a tantalum crucible maintained at 80 ºC and 200 ºC, respectively. After annealing step, the sample was transferred to the STM stage held at 4.2 K. Conductance d*I*/d*V* point spectra and maps were acquired with conventional lock-in technique with a modulation of 1 and 10 mV, respectively (at frequency of 952 Hz). All data were subject of standard processes using the WSxM software[1].

For data analysis approximately 100 polymers were considered in dozens of STM overviews of 50 x 50 nm^2. The bond-length analysis considered a total set of 12 cis- and trans- monomers.

DFT calculations

A. dI/dV simulations in the gas phase

Density functional theory (DFT) calculations were carried out using the all electron FHI-AIMS code[2]. For the description of the electronic structure of the freestanding unit cell, the hybrid exchange-correlation functional B3LYP[3] was employed in the calculations. The freestanding unit cell consists of 44 atoms. In all calculations, the default light settings were used for the numerical atomic basis sets. In all total energy calculations, the atomic structures were thoroughly relaxed until the Hellman-Feynman forces were smaller than 10^{-2} eV $Å^{-1}$. To sample the Brillouin zone, all geometrical optimizations of the infinite systems with a one-dimensional periodic boundary condition used a Monkhorst-Pack grid of 18 x 1 x 1 k-points. The band structure plots were obtained with 50 k-points along the direction of 1D periodicity.

Theoretical d*I*/d*V* maps were calculated by the Probe Particle Scanning Tunneling Microscopy (PPSTM) code[4] for a CO-like orbital tip, represented by a combination of P_xP_y (90%) and s-like wave character (10%) without tip relaxation.

B. AFM Simulations

The AFM images were calculated with Probe Particle code[5] using the effective lateral stiffness k = 0.7 N/m for the flexible probe-particle tip model. The electrostatic interaction between the molecule and the probe particle was calculated using Hartree potential of polymer and differential electron density of CO-tip obtained from fully optimized DFT calculations. Pauli repulsion was calculated from the total density of both polymer and CO-tip.

C. Aromaticity

Gaussian16[6] program was used for the ACID and NICS calculations with ωB97X-D[7] hybrid functional and def2-SVP[8] basis set. NICS values were calculated 1 Å above molecular plane and only zz component was gathered. Only p_z orbitals were included in the ACID calculations.

Figures S1 to S8

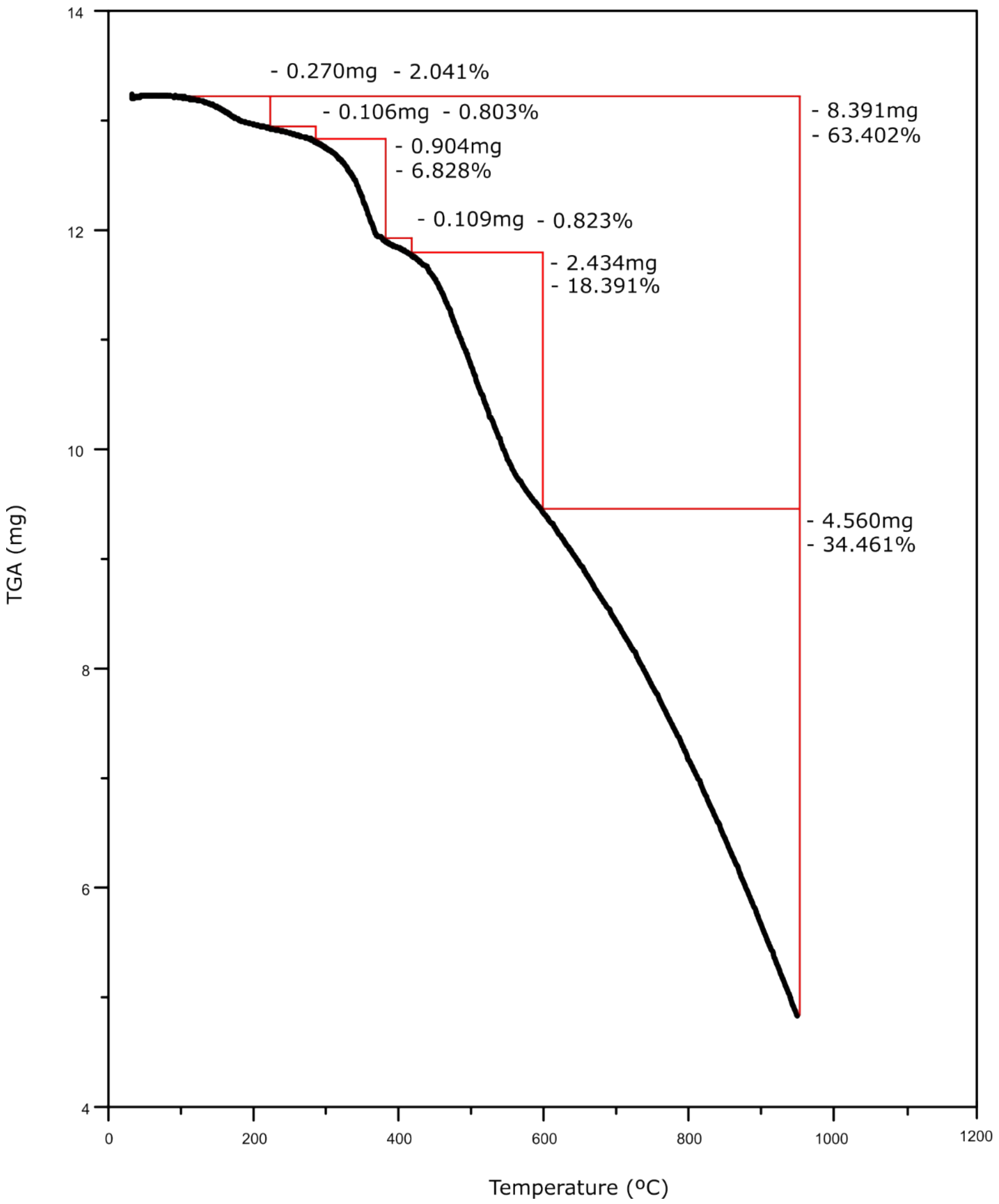

Figure S1. Thermogravimetric Analysis of compound 4. Analysis done under N_2 atmosphere showing that there is no significant change in molecule 4 at temperatures below of its sublimation temperature.

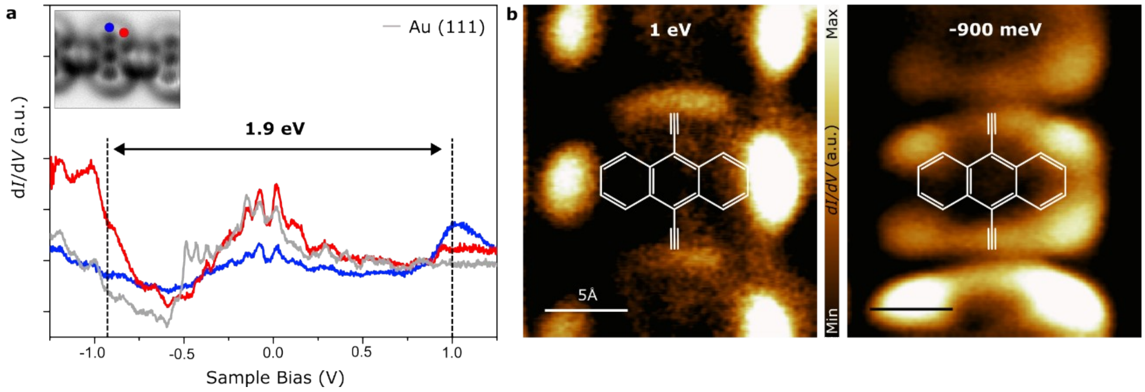

Figure S2. Electronic structure of diethynyl-anthracene polymer (2) on Au(111). a) dI/dV point spectra at the positions of the circles in the inset. In gray color spectra taken on the Au substrate for comparison. b) Conductance maps acquired at 1 eV and at -0.9 eV with a superimposed model of the anthracene monomer.

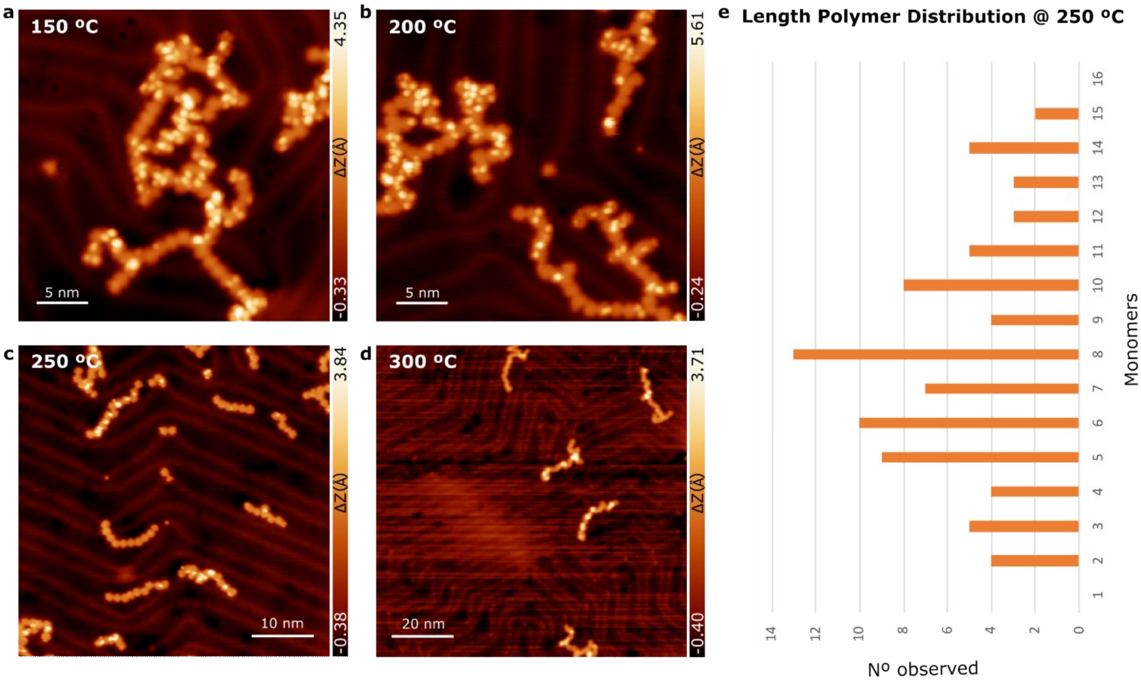

Figure S3. Growth of polymer (7) *vs* temperature. STM overview image of the sample after annealing at a) 150 ºC (0.5 V, 0.01 nA), b) 200 ºC (1 V, 0.02 nA), c) 250 ºC (0.1 V, 0.02 nA), d) 300 ºC (0.1 V, 0.01 nA). e) Length distribution of the polymers at 250 ºC.

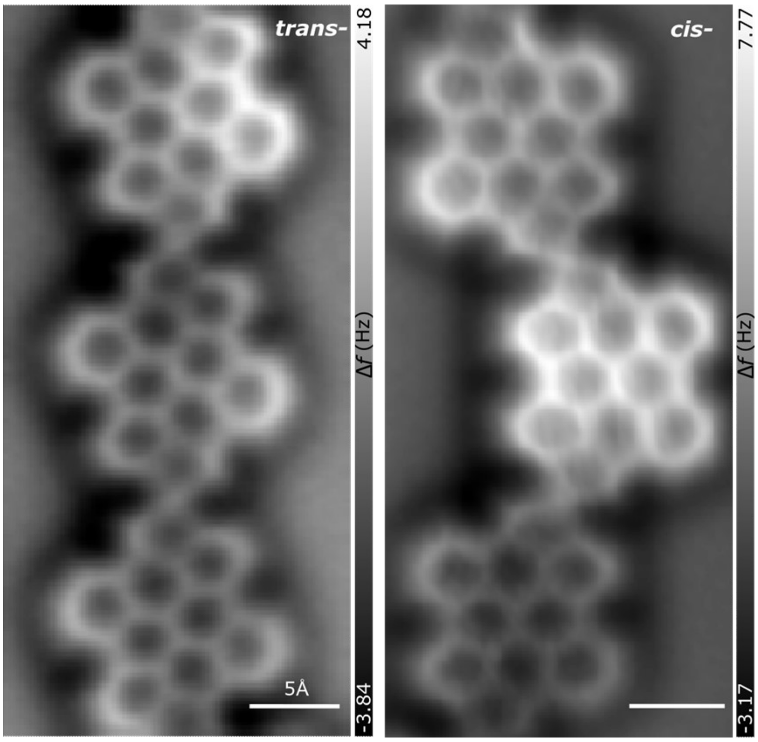

Figure S4. Trans-/Cis- isomerism of bisanthene-fulvalene polymers. High-resolution nc-AFM images of trans- and cis- configurations that can be adopted by the polymer.

2 **5**

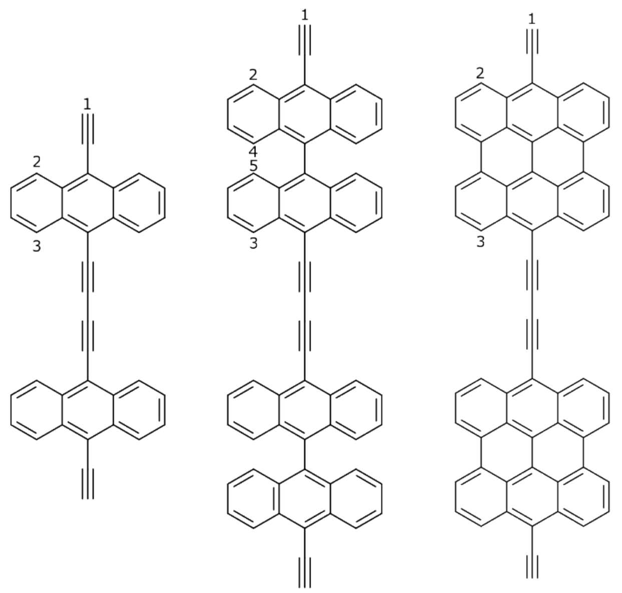

BDE (kcal/mol)	Anthracene (2)	Bianthracene (5)	Bisanthene
1	120.3	99	109.1
2	118.6	114.1	117.4
3	118.5	113.3	118.7
4	-	110.8	-
5	-	111.9	-

Figure S5. Bond dissociation energy analysis. Bond dissociation energy values of the reaction-participant radical positions for anthracene (2), bianthracene (5), and bisanthene polymers in gas phase.

a

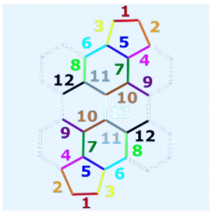

Bond # / Å	1	2	3	4	5	6	7	8	9	10	11	12	MEAN
1	1.45	1.45	1.47	1.47	1.48	1.47	1.44	1.43	1.47	1.45	1.48	1.47	1.46(2)
2	1.47	1.47	1.47	1.46	1.45	1.46	1.45	1.45	1.46	1.45	1.46	1.44	1.45(1)
3	1.35	1.36	1.34	1.34	1.35	1.35	1.33	1.35	1.35	1.33	1.35	1.34	1.35(1)
4	1.42	1.42	1.41	1.4	1.41	1.4	1.4	1.4	1.43	1.43	1.37	1.39	1.41(2)
5	1.48	1.48	1.42	1.42	1.45	1.44	1.47	1.46	1.45	1.46	1.46	1.47	1.46(2)
6	1.46	1.46	1.43	1.43	1.43	1.41	1.43	1.41	1.43	1.41	1.41	1.43	1.43(2)
7	1.41	1.41	1.35	1.35	1.36	1.36	1.35	1.34	1.34	1.32	1.35	1.34	1.36(3)
8	1.42	1.42	1.44	1.45	1.42	1.45	1.43	1.43	1.45	1.43	1.43	1.43	1.43(1)
9	1.43	1.43	1.43	1.43	1.4	1.39	1.41	1.42	1.42	1.42	1.44	1.43	1.42(1)
10	1.46	1.46	1.43	1.43	1.41	1.41	1.44	1.46	1.46	1.45	1.43	1.42	1.44(2)
11	1.47	1.47	1.46	1.47	1.43	1.45	1.45	1.45	1.43	1.45	1.43	1.45	1.45(2)
12	1.41	1.41	1.41	1.4	1.44	1.42	1.43	1.44	1.44	1.47	1.42	1.42	1.43(2)
13	1.38	1.38	1.38	1.38	1.36	1.36	1.38	1.38	1.33	1.33	1.35	1.35	1.36(2)

(Monomer # across columns)

b

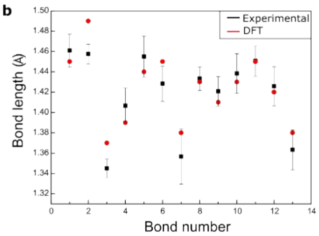

c

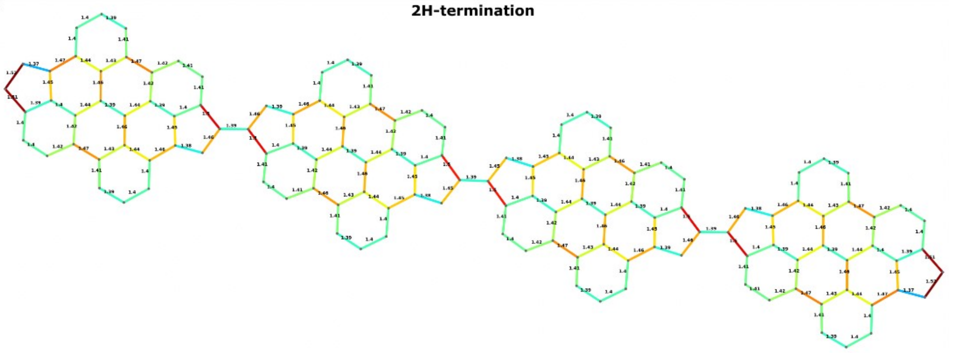

d

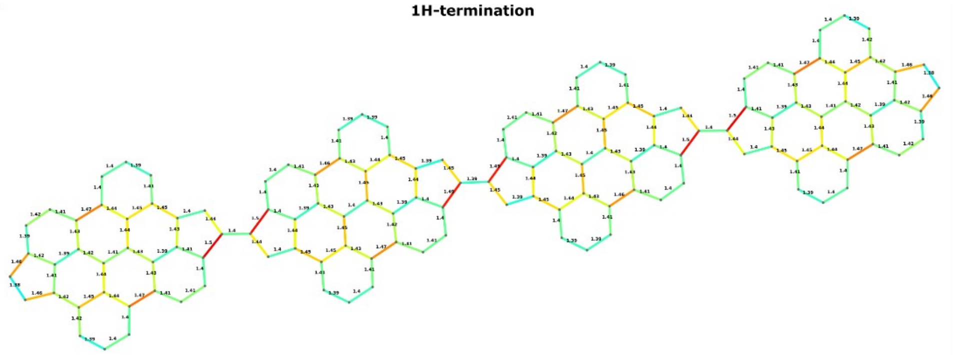

Figure S6. Bond length analysis of bisanthene fulvalene-bridged polymers (7). a) Bisanthene monomer model with colours and numbers of labels. Table with the observed bond lengths. b) Plot of the experimental (black) and the DFT (red) bond length values. c,d) Bond length values for c) 2H-termination; d) 1H-termination fulvalene-bonded bisanthene tetramer polymer.

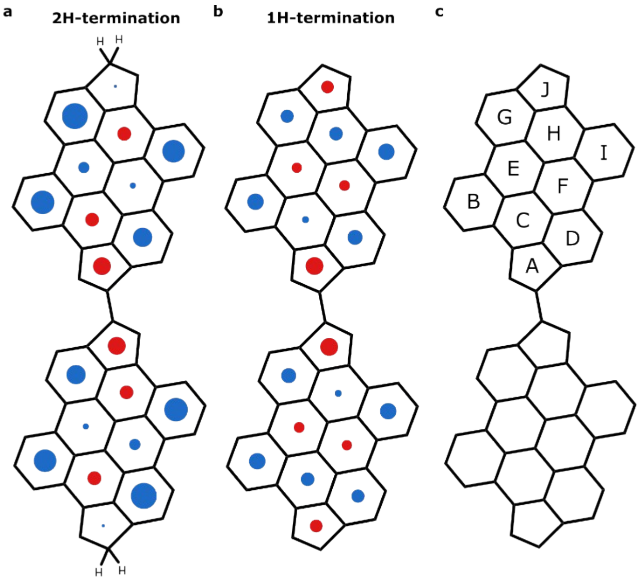

	2H	1H
A	15.4	15.2
B	-21.0	-14.6
C	11.7	-6.5
D	-16.2	-13.5
E	-9.8	9.0
F	-4.3	9.7
G	-23.8	-11.3
H	11.5	-11.7
I	-20.7	-14.2
J	-2.0	11.3

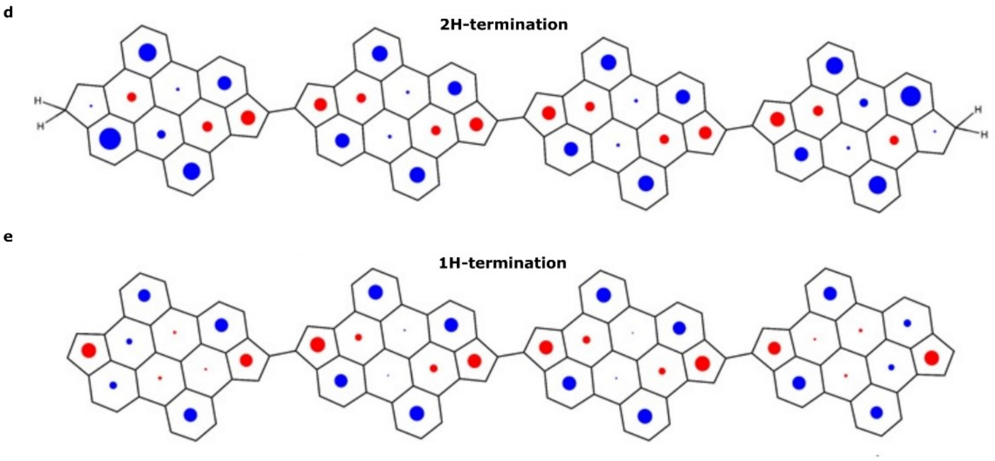

Figure S7. NICS analysis of fulvalene-linked dime and tetramer. In blue/red negative/positive NICS values of a) 2H-terminated and b) 1H-terminated dimer. c) Table with all NICS values. Diameter of the circular feature at the centre of the six- and five-membered rings directly corresponds to the NICS value. NICS representation image of d) 2H and e) 1H terminated tetramer, respectively.

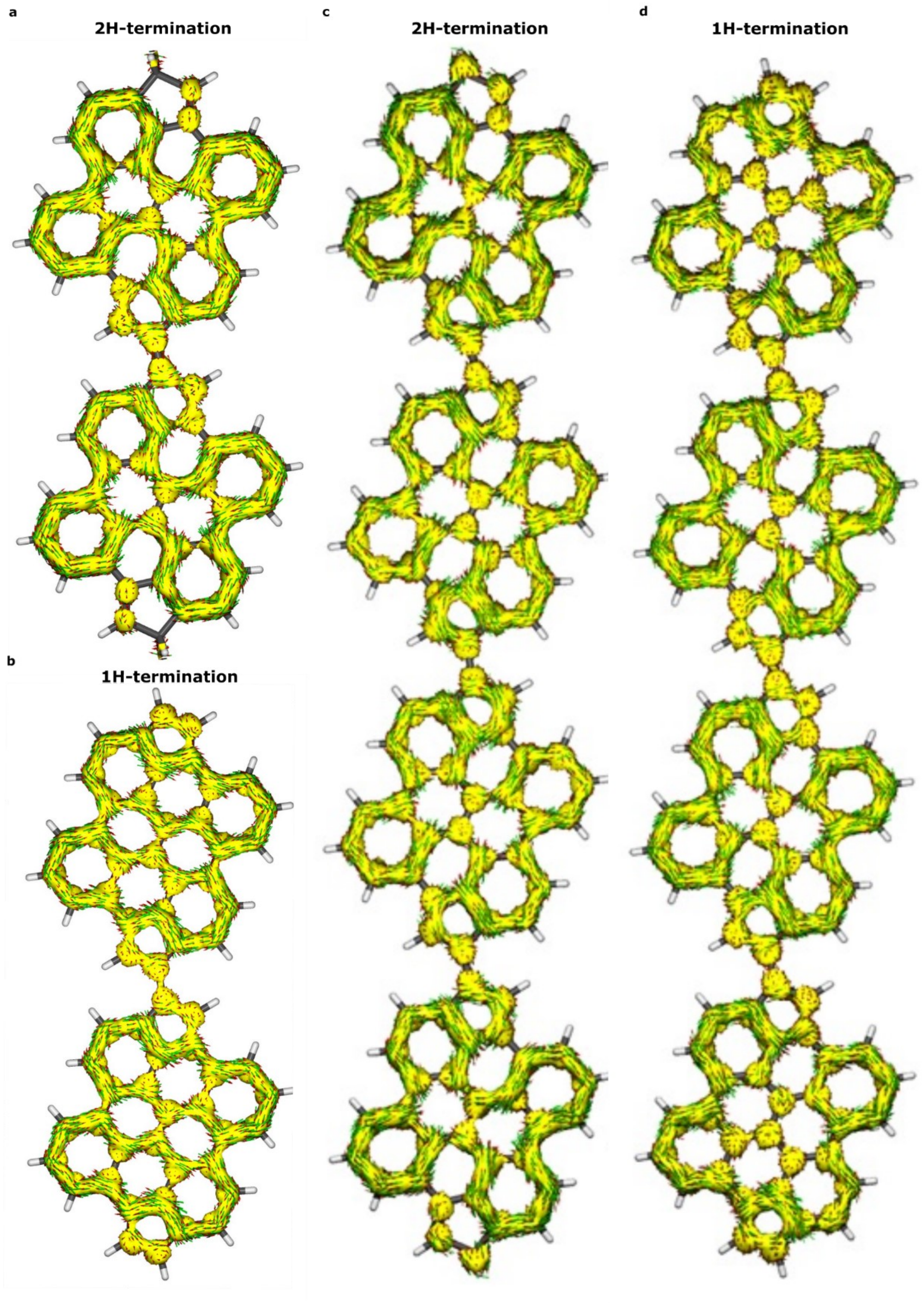

Figure S8. ACID analysis of fulvalene-linked dimer. ACID image with imposed current density inside a dimer when a: a) 2H-termination; b) 1H-termination is considered as the end of the polymer. ACID calculations of a tetramer when c) 2H-termination; d) 1H-termination of the fulvalene-bonded polymer. The similar aromaticity behaviour for both cases indicates the preference of double-bond fulvalene-linker.

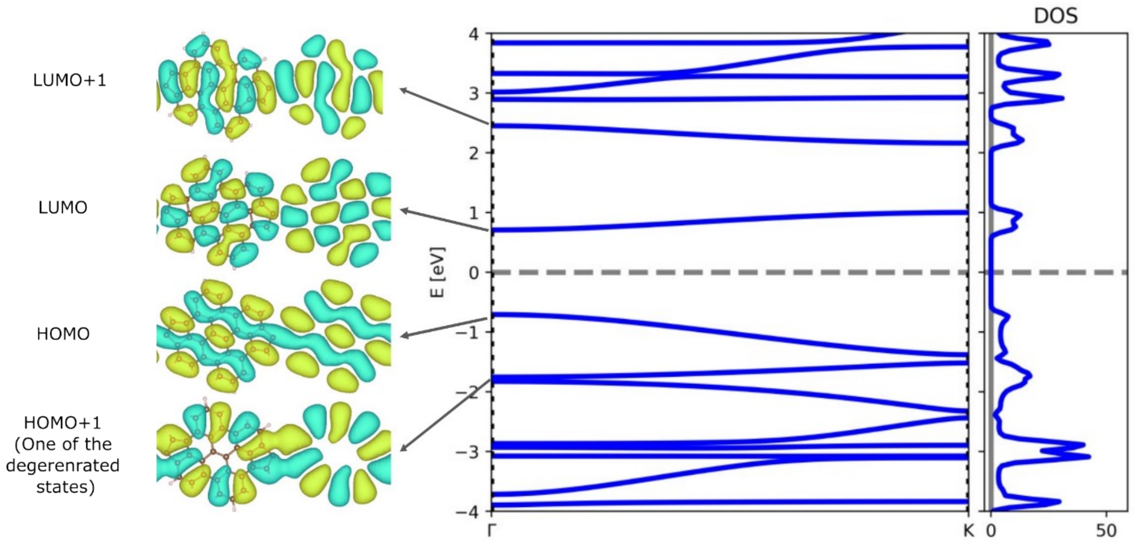

Figure S9. DFT electronic structure of the fulvalene polymer. a) DFT calculated canonical molecular orbitals. b) Projected density of states.

Synthesis of the materials

Synthesis Overview

Compound 1:

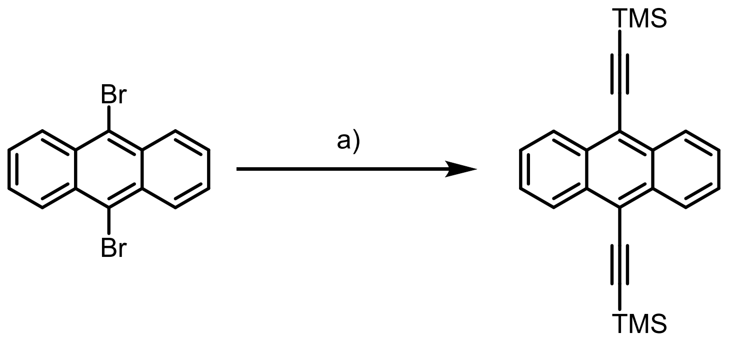

S1 **1**

Scheme S1. Synthesis of compound **1**. Reagents and conditions: a) $PdCl_2(CH_3CN)_2$, CuI, $P(^tBu)_3H{\cdot}BF_4$, ^{t}BuNH$_2$, Trimethylsilylacetylene, THF, $(^iPr)_2NH$, r.t., 16 h, 91%.

Compound 4:

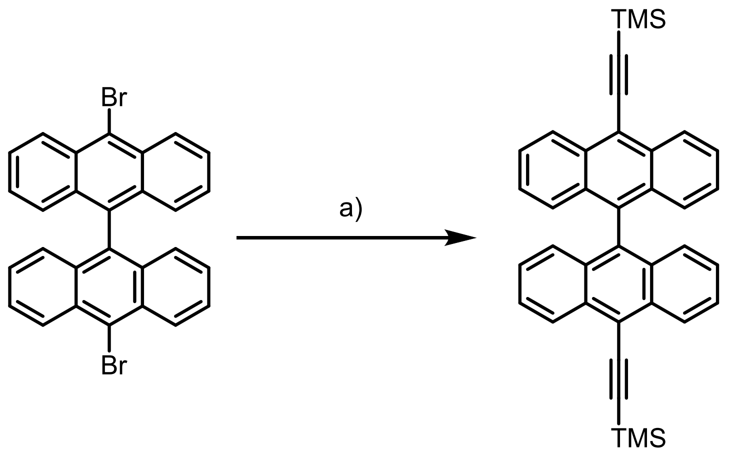

S2 **4**

Scheme S2. Synthesis of compound **4**. Reagents and conditions: a) $PdCl_2(CH_3CN)_2$, CuI, $P(^tBu)_3H{\cdot}BF_4$, ^{t}BuNH$_2$, Trimethylsilylacetylene, THF, $(^iPr)_2NH$, r.t., 16 h, 84%.

Synthesis of molecular precursors

Unless otherwise noted, commercially available reagents, solvents and anhydrous solvents were used as purchased without further purification. Dry THF was freshly distilled over Na/benzophenone. TLC plates were purchased from Sigma-Aldrich (silica gel matrix, with fluorescent indicator 254 nm) and were stained with phosphomolybdic acid (5% ethanol solution), or observed under UV light. Flash column chromatography was performed with Silica gel 60 (230-400 mesh, Scharlab, Spain). Silica gel G

preparative TLC plates (20x20 cm, 500 micron) were purchased from ANALTECH. ^{1}H and ^{13}C NMR spectra were recorded at room temperature on a Bruker Avance and referenced to the signal of the residual protiated solvent (^{1}H: $\delta = 7.26$ for $CDCl_3$ at room temperature) or the ^{13}C signal of the solvents (^{13}C: δ = 77.16 for $CDCl_3$) or to the signal of the residual TMS (^{1}H: δ = 0.00). Coupling constant (J) values are given in Hz. Abbreviations indicating multiplicity were used as follow: t = triplet, d = doublet, s = singlet. Assignment of the ^{13}C NMR multiplicities was accomplished by DEPT techniques. MALDI-TOF mass spectra were recorded on a Bruker Ultraflex III mass spectrometer. IR spectra were recorded with a Perkin-Elmer Spectrum Two FTIR ATR spectrometer.

Synthesis procedures and characterization details

Compound 1:

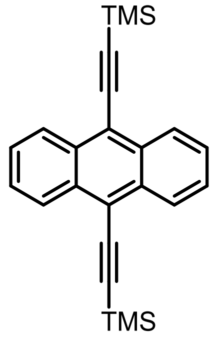

In a round bottom flask, 9,10-Dibromoanthracene (500 mg, 1.49 mmol), $PdCl_2(CH_3CN)_2$ (65 mg, 0,22 mmol), CuI (85 mg, 0,45 mmol), $P(^{t}Bu)_3H{\cdot}BF_4$ (130 mg, 0.45 mmol) were placed and purged with Ar. The solids were supendend in THF (4 mL), $(^{i}Pr)_2NH$ (10 mL) was added and the suspension bubbled with Ar. Trimethylsilylacetylene (0.63 mL, 4.46 mmol) was added dropwise and the reaction mixture was stirred at room temperature for 16h. The solvent was removed under reduced pressure, diluted with CH_2Cl_2 and washed with NH_4Cl_{sat}. The organic layer was dried over anhydrous Na_2SO_4, filtered and the crude purified by flash column chromatography (SiO_2, hexane) affording **1** (502 mg, 91%) as a yellow powder.

^{1}H NMR (400 MHz, $CDCl_3$) δ 8.58 (dd, *J* = 6.8, 3.3 Hz, 4H), 7.61 (dd, *J* = 6.9, 3.3 Hz, 4H), 0.43 (s, 2H) Spectral data agree with those previously reported[9].

Compound 4:

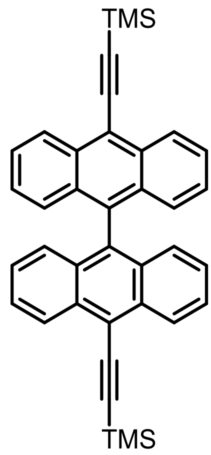

In a round bottom flask, 10,10′-dibromo-9,9′-bianthracene (500 mg, 0.98 mmol), $PdCl_2(CH_3CN)_2$ (42 mg, 0,15 mmol), CuI (56 mg, 0,29 mmol), $P(^tBu)_3H\cdot BF_4$ (85mg, 0.293 mmol) were placed and purged with Ar. The solids were supendend in THF (3 mL), $(^iPr)_2NH$ (7 mL) was added and the suspension bubbled with Ar. Trimethylsilylacetylene (0.41 mL, 2.93 mmol) was added dropwise and the reaction mixture was stirred at room temperature for 16h. The solvent was removed under reduced pressure, diluted with CH_2Cl_2 and washed with NH_4Cl_{sat}. The organic layer was dried over anhydrous Na_2SO_4, filtered and the crude purified by flash column chromatography (SiO_2, hexane/CH_2Cl_2 95:5) affording **1** (449 mg, 84%) as a yellow powder.

^{1}H NMR (500 MHz, $CDCl_3$) δ 8.74 (d, J = 8.7 Hz, 4H), 7.56 (t, J = 7.6 Hz, 4H), 7.20 – 7.14 (t, J = 8.7 Hz, 4H), 7.07 (d, J = 8.7 Hz, 4H), 0.50 (s, 18H). **^{13}C NMR** (126 MHz, $CDCl_3$) δ 134.53, 132.77, 131.22, 127.29, 127.27, 126.84, 126.40, 118.32, 107.27, 101.82, 0.46. **HR-MS (MALDI-TOF):** m/z calc. for $C_{38}H_{34}Si_2$ $[M]^+$: 546.2194, found 546.2188. **IR (ATR):** 2957, 2136, 1437, 1345, 1249 cm^{-1}.

NMR spectra

Compound 4:

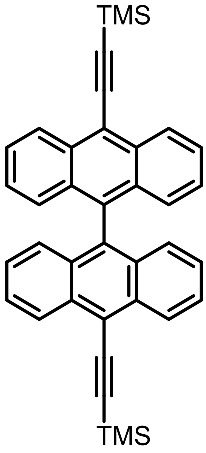

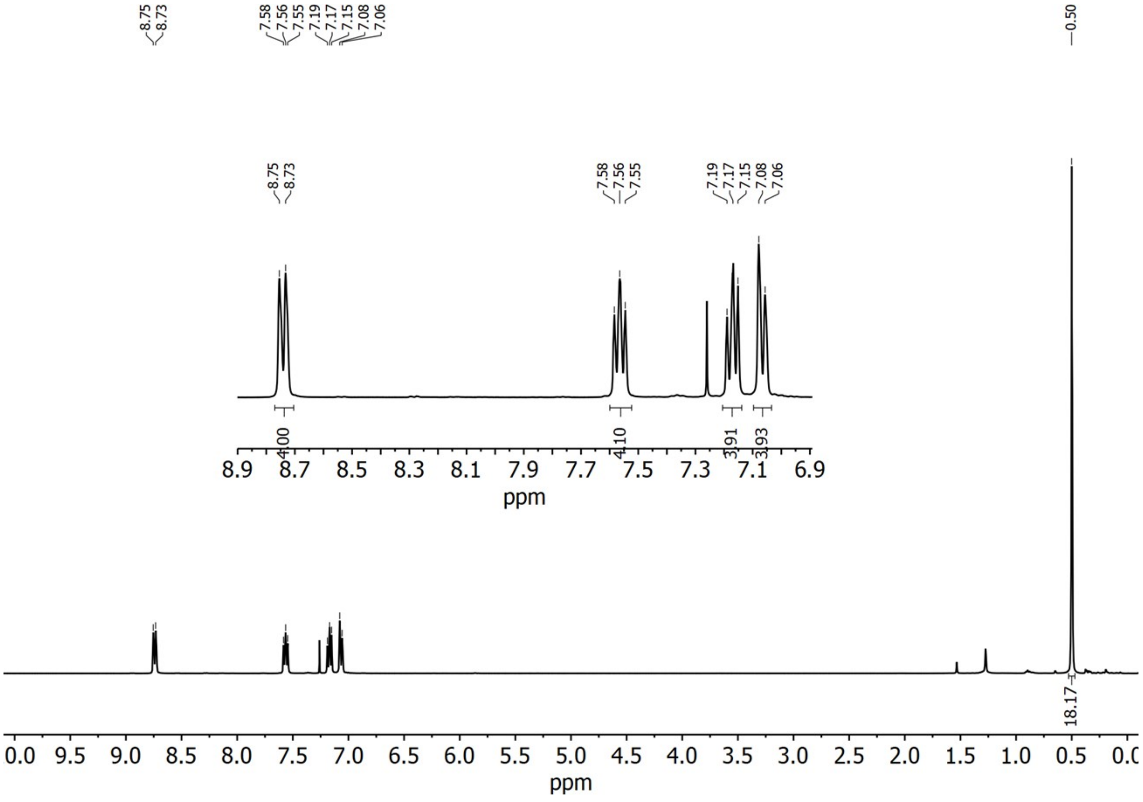

Figure S9. ^{1}H NMR (500 MHz, $CDCl_3$) spectrum of **4**.

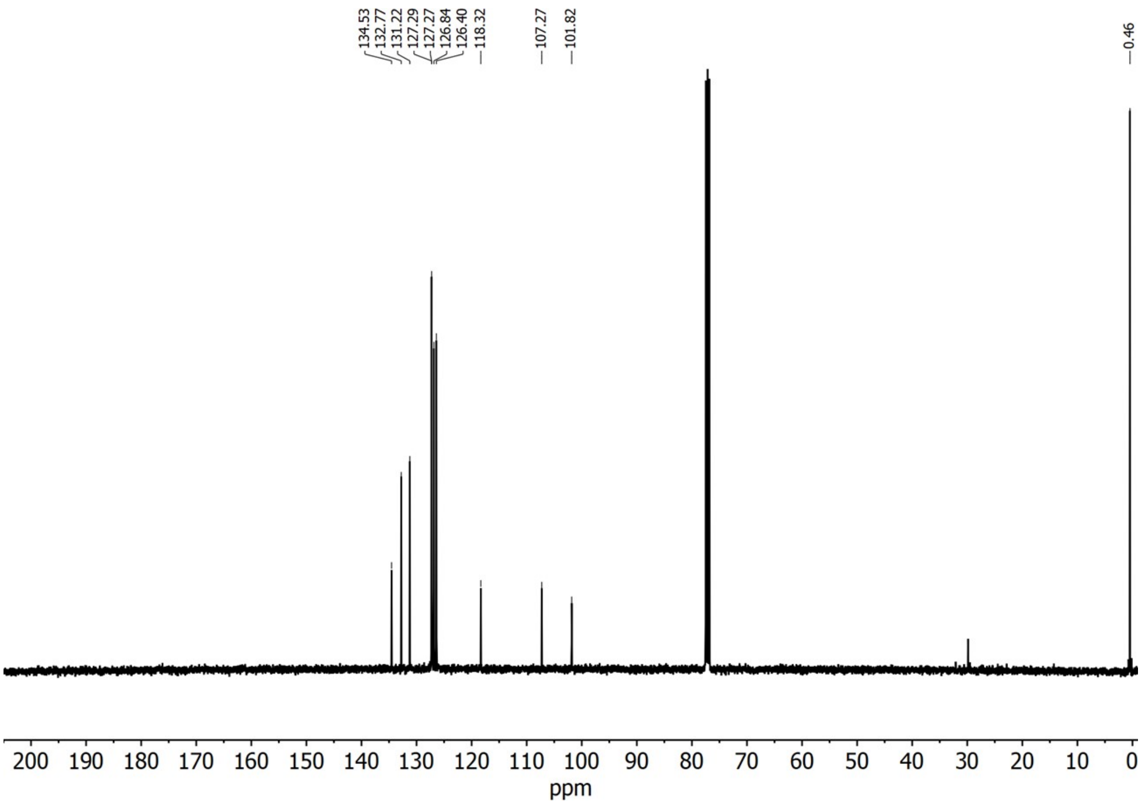

Figure S10: ^{13}C NMR (126 MHz, $CDCl_3$) spectrum of **4**.

MS spectra

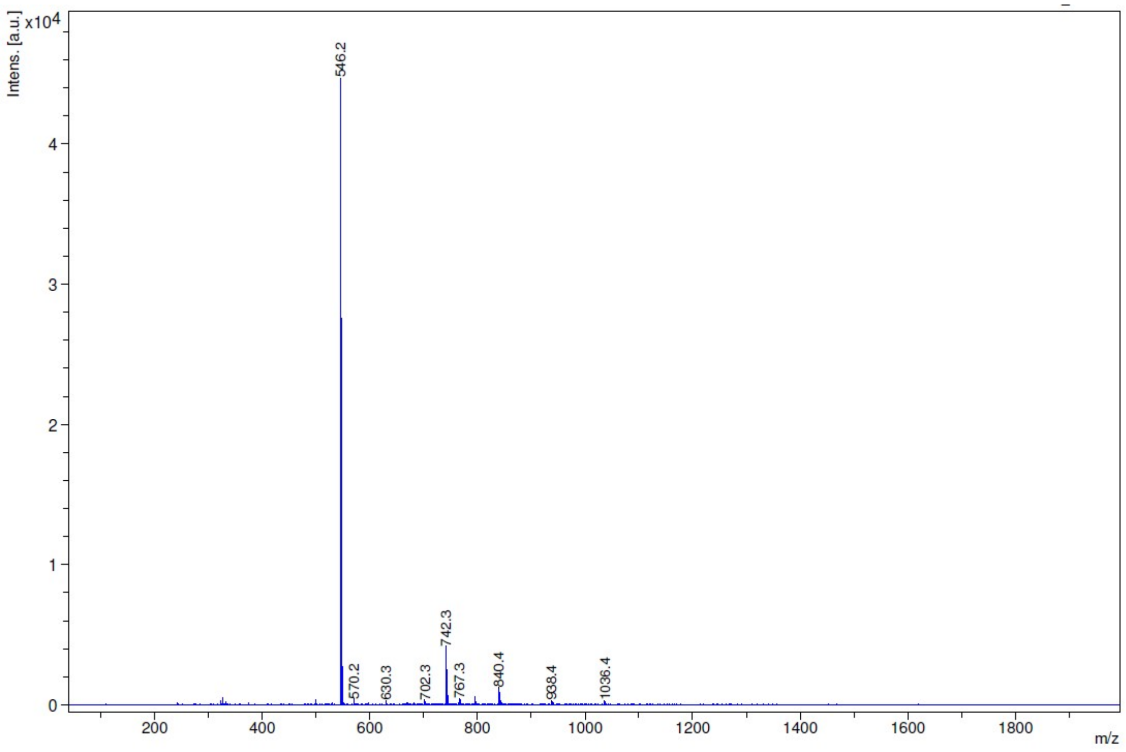

Figure S11. MS (MALDI$^+$) spectrum of **4**.

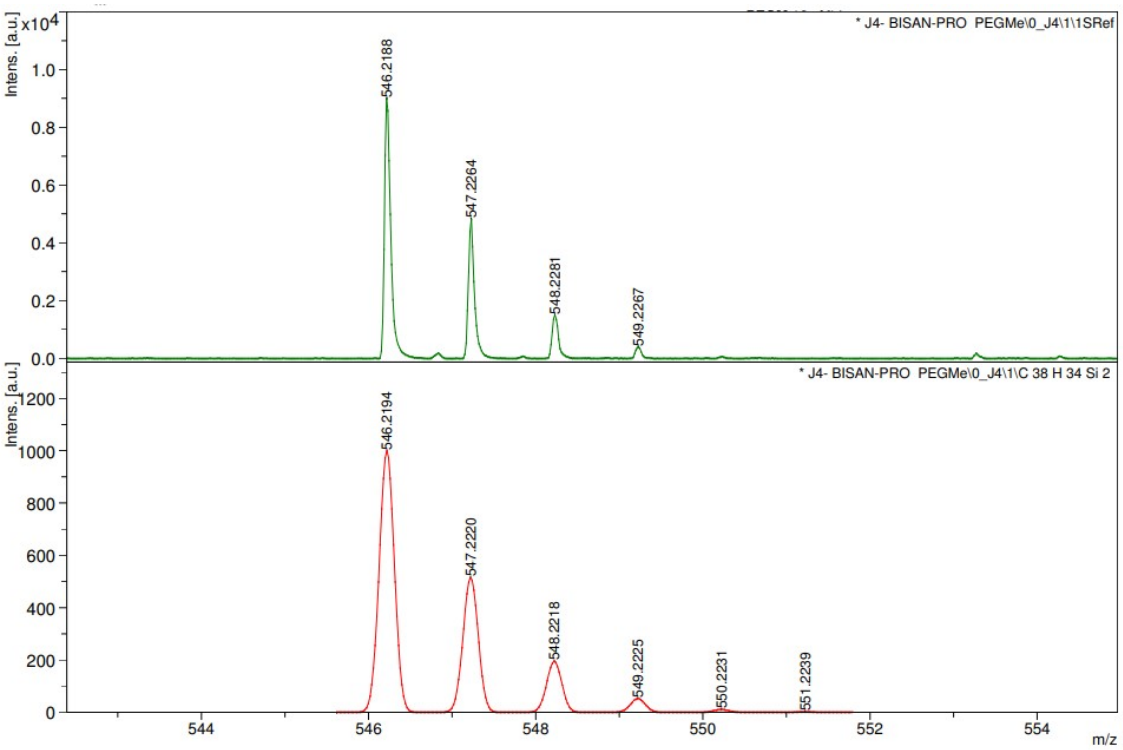

Figure S12. HR-MS (MALDI-TOF) Observed (bottom) and calculated (top) isotopic distribution for **4**.

Single crystal X-Ray crystallography

X-ray diffraction-quality single crystals of compound **4** were obtained by vapor diffusion of acetonitrile into a chloroform solution of **4**. The X-ray diffraction measurement was carried out on a Bruker D8 Venture diffractometer with a Photon III detector using Mo Kα radiation. SHELXT[10] was used to solve the structure, which was then refined applying the full-matrix least-squares against F^2 procedure with SHELX 2018[11] using the WinGX32[12] software. During the refinement, hydrogen atoms were placed in idealized positions (U_{eg}(H) = 1.2U_{eg}(C) or U_{eg}(H) = 1.5U_{eg}(C)) and were allowed to ride on their parent atoms.

Summary of the X-ray diffraction measurement and refinement data: Chemical formula, $C_{38}H_{34}Si_2$; *Mr*, 546.38; crystal size [mm^3], 0.300 × 0.230 × 0.030; temperature, 100(2) K; wavelength [Å], 0.71073 (Mo Kα), crystal system, orthorhombic; space group, *Pbca*; *a* [Å], 11.633(4); *b* [Å], 13.377(4); *c* [Å], 41.461(15); α [°], 90; β [°], 90; γ [°], 90; *V* [Å^3], 6452(4); *Z*, 8; ρ_{calcd} [Mg m^{-3}], 1.126; μ [mm^{-1}], 0.134; F(000), 2320; θ range [°], 1.965 to 18.848; *hkl* ranges, −10/10, −12/12, −26/37; reflections collected, 19189; independent reflections, 2525; R_{int}, 0.1001; completeness to θ = 18.848°, 99.6%; absorption correction, semi-empirical from equivalents; refinement method; full-matrix least-squares on F^2; Final *R* indices [I>2σ(I)], R_1 = 0.0661, wR_2 = 0.1716; *R* indices (all data), R_1 = 0.0865, wR_2 = 0.1876; goodness-of-fit on F^2, 1.034.

CCDC-2193258 contains the supplementary crystallographic data for this paper. These data can be obtained free of charge from The Cambridge Crystallographic Data Centre via https://www.ccdc.cam.ac.uk/structures/

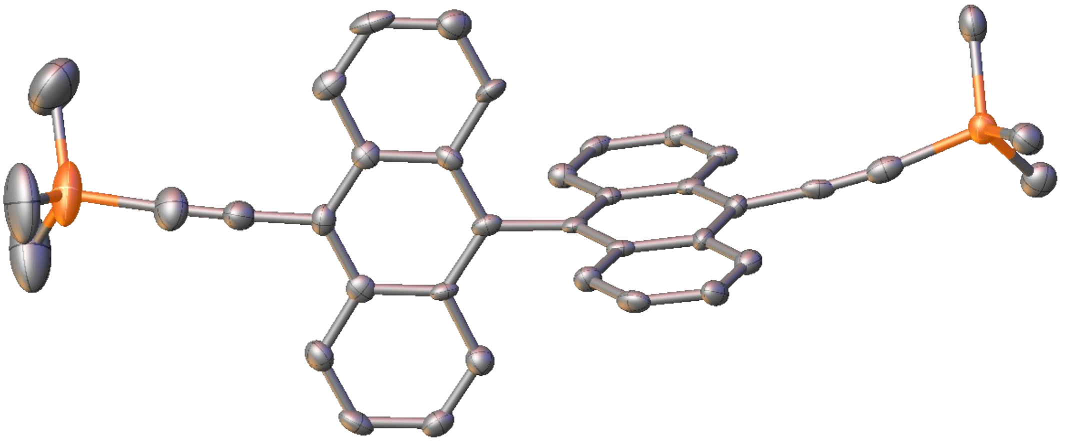

Fig. S13. ORTEP-type[13] drawing of the structure of compound **4**, showing the thermal ellipsoids at 50% probability. H atoms have been omitted for clarity.

References

(1) Horcas, I.; Fernández, R.; Gómez-Rodríguez, J. M.; Colchero, J.; Gómez-Herrero, J.; Baro, A. M. WSXM: A Software for Scanning Probe Microscopy and a Tool for Nanotechnology. *Review of Scientific Instruments* **2007**, *78* (1). https://doi.org/10.1063/1.2432410.

(2) Blum, V.; Gehrke, R.; Hanke, F.; Havu, P.; Havu, V.; Ren, X.; Reuter, K.; Scheffler, M. Ab Initio Molecular Simulations with Numeric Atom-Centered Orbitals. *Computer Physics Communications* **2009**, *180* (11), 2175–2196. https://doi.org/10.1016/J.CPC.2009.06.022.

(3) Becke, A. D. A New Mixing of Hartree–Fock and Local Density-functional Theories. *The Journal of Chemical Physics* **1998**, *98* (2), 1372. https://doi.org/10.1063/1.464304.

(4) Krejčí, O.; Hapala, P.; Ondráček, M.; Jelínek, P. Principles and Simulations of High-Resolution STM Imaging with a Flexible Tip Apex. *Physical Review B* **2017**, *95* (4), 045407. https://doi.org/10.1103/PHYSREVB.95.045407/FIGURES/6/MEDIUM.

(5) Hapala, P.; Kichin, G.; Wagner, C.; Tautz, F. S.; Temirov, R.; Jelínek, P. Mechanism of High-Resolution STM/AFM Imaging with Functionalized Tips. *Physical Review B - Condensed Matter and Materials Physics* **2014**, *90* (8), 085421. https://doi.org/10.1103/PHYSREVB.90.085421/FIGURES/6/MEDIUM.

(6) Frisch, M. J.; Trucks, G. W.; Schlegel, H. B.; Scuseria, G. E.; Robb, M. A.; Cheeseman, J. R.; Scalmani, G.; Barone, V.; Petersson, G. A.; Nakatsuji, H.; Li, X.; Caricato, M.; Marenich, A. v.; Bloino, J.; Janesko, B. G.; Gomperts, R.; Mennucci, B.; Hratchian, H. P.; Ortiz, J. v.; Izmaylov, A. F.; Sonnenberg, J. L.; Williams-Young, D.; Ding, F.; Lipparini, F.; Egidi, F.; Goings, J.; Peng, B.; Petrone, A.; Henderson, T.; Ranasinghe, D.; Zakrzewski, V. G.; Gao, J.; Rega, N.; Zheng, G.; Liang, W.; Hada, M.; Ehara, M.; Toyota, K.; Fukuda, R.; Hasegawa, J.; Ishida, M.; Nakajima, T.; Honda, Y.; Kitao, O.; Nakai, H.; Vreven, T.; Throssell, K.; Montgomery, J. A.; Jr.; Peralta, J. E.; Ogliaro, F.; Bearpark, M. J.; Heyd, J. J.; Brothers, E. N.; Kudin, K. N.; Staroverov, V. N.; Keith, T. A.; Kobayashi, R.; Normand, J.; Raghavachari, K.; Rendell, A. P.; Burant, J. C.; Iyengar, S. S.; Tomasi, J.; Cossi, M.; Millam, J. M.; Klene, M.; Adamo, C.; Cammi, R.; Ochterski, J. W.; Martin, R. L.; Morokuma, K.; Farkas, O.; Foresman, J. B.; Fox, D. J. Gaussian 16, Rev. C.01. *Gaussian 16, Rev. C. 01*. 2016.

(7) Chai, J. da; Head-Gordon, M. Long-Range Corrected Hybrid Density Functionals with Damped Atom–Atom Dispersion Corrections. *Physical Chemistry Chemical Physics* **2008**, *10* (44), 6615–6620. https://doi.org/10.1039/B810189B.

(8) Weigend, F.; Ahlrichs, R. Balanced Basis Sets of Split Valence, Triple Zeta Valence and Quadruple Zeta Valence Quality for H to Rn: Design and Assessment of Accuracy. *Physical Chemistry Chemical Physics* **2005**, *7* (18), 3297. https://doi.org/10.1039/b508541a.

(9) Fudickar, W.; Linker, T. Why Triple Bonds Protect Acenes from Oxidation and Decomposition. *J Am Chem Soc* **2012**, *134* (36), 15071–15082. https://doi.org/10.1021/JA306056X/SUPPL_FILE/JA306056X_SI_001.PDF.

(10) Sheldrick, G. M.; IUCr. SHELXT – Integrated Space-Group and Crystal-Structure Determination. *urn:issn:2053-2733* **2015**, *71* (1), 3–8. https://doi.org/10.1107/S2053273314026370.

(11) Sheldrick, G. M. A Short History of *SHELX*. *Acta Crystallographica Section A Foundations of Crystallography* **2008**, *64* (1), 112–122. https://doi.org/10.1107/S0108767307043930.

(12) Farrugia, L. J.; IUCr. WinGX and ORTEP for Windows: An Update. *urn:issn:0021-8898* **2012**, *45* (4), 849–854. https://doi.org/10.1107/S0021889812029111.

(13) Dolomanov, O. v.; Bourhis, L. J.; Gildea, R. J.; Howard, J. A. K.; Puschmann, H. OLEX2: A Complete Structure Solution, Refinement and Analysis Program. *urn:issn:0021-8898* **2009**, *42* (2), 339–341. https://doi.org/10.1107/S0021889808042726.
